# Hydrogen Dissociation Reaction on First-Row Transition Metal Doped Nanobelts

**DOI:** 10.3390/ma16072792

**Published:** 2023-03-31

**Authors:** Imene Bayach, Sehrish Sarfaraz, Nadeem S. Sheikh, Kawther Alamer, Nadiah Almutlaq, Khurshid Ayub

**Affiliations:** 1 Department of Chemistry, College of Science, King Faisal University, Al-Ahsa 31982, Saudi Arabia; 2 Department of Chemistry, COMSATS University Islamabad, Abbottabad Campus, Abbottabad 22060, Pakistan; 3 Chemical Sciences, Faculty of Science, Universiti Brunei Darussalam, Jalan Tungku Link, Gadong BE1410, Brunei

**Keywords:** molecular nanobelts, transition metal, dissociation barrier, density functional theory, hydrogen molecule

## Abstract

Zigzag molecular nanobelts have recently captured the interest of scientists because of their appealing aesthetic structures, intriguing chemical reactivities, and tantalizing features. In the current study, first-row transition metals supported on an H_6_-N_3_-belt[6]arene nanobelt are investigated for the electrocatalytic properties of these complexes for the hydrogen dissociation reaction (HDR). The interaction of the doped transition metal atom with the nanobelt is evaluated through interaction energy analysis, which reveals the significant thermodynamic stability of TM-doped nanobelt complexes. Electronic properties such as frontier molecular orbitals and natural bond orbitals analyses are also computed, to estimate the electronic perturbation upon doping. The highest reduction in the HOMO–LUMO energy gap compared to the bare nanobelt is seen in the case of the Zn@NB catalyst (4.76 eV). Furthermore, for the HDR reaction, the Sc@NB catalyst displays the best catalytic activity among the studied catalysts, with a hydrogen dissociation barrier of 0.13 eV, whereas the second-best catalytic activity is observed for the Zn@NB catalyst (0.36 eV). It is further found that multiple active sites, i.e., the presence of the metal atom and nitrogen atom moiety, help to facilitate the dissociation of the hydrogen molecule. These key findings of this study enhance the understanding of the relative stability, electronic features, and catalytic bindings of various TM@NB catalysts.

## 1. Introduction

With advancements in modern society, hydrogen is predominantly viewed as “the fuel of the future”, as well as an eminent energy carrier, due to its clean, green, and ecofriendly nature [1]. Hydrogen technology has wide ranging applications; however, its practical implementations need to be acquired yet [2]. One of the key barriers in the implementation of hydrogen technology is an effective and low-cost hydrogen storage process [3]. The hydrogen dissociation reaction (HDR) in this regard is considered as the principle step in the hydrogen storage process. The main problem with the feasible hydrogen economy is the hydrogen storage, and so far, searching for a cost-effective strategy of storing hydrogen is considered as an indomitable challenge. Scientists are trying to search for innovative ways that can better help to store hydrogen. In recent times, hydrogen can be stored as liquid hydrogen, compressed hydrogen, and as a storage material [4]. The capture and discharge of H_2_ on materials involves the process of molecular adsorption, chemical bonding, diffusion, and dissociation [5,6]. Moreover, the hydrogen splitting reaction over various metal surfaces is often grasped as a prototype system for the examination of the gas–catalyst interaction, and thus, an understanding of the catalytic essence due to a simple reaction mechanism [7]. 

Additionally, other reactions involving HDR include the catalytic hydrogenation reaction, which is an important step in several industrial processes. The hydrogen dissociation reaction (HDR) is a key step in almost all hydrogenation processes that are central to pollutant removal and industrial chemical production [7]. Moreover, the hydrotreating process also requires the activation of a hydrogen molecule on the catalyst surface, and subsequent reactions between adsorbed hydrogen and other organic species [8]. In the catalytic world, these hydrogenation reactions display a vital contribution to industrial chemical processes with respect to market sales [9]. Metal-decorated electrocatalysts have been abundantly utilized as catalysts due to their appropriate electronic structures, which allows for the easy adsorption and dissociation of the H_2_ molecule on the metal catalytic surface [10,11,12]. 

Platinum group noble metal-based materials have been considered as the most effective catalysts in energy production application, and they have surpassed all conventional catalysts [13]. However, it is still necessary to design low-platinum or non-platinum catalysts, due to the expensiveness and rareness of platinum [14,15]. In this regard, the non-precious metal catalysts, which usually consist of metal elements with high relative abundances, such as Mn, Fe, Cu, Ni, etc., have shown a catalytic performance that is almost comparable with Pt [16,17,18]. Significant research efforts have been displayed in recent years to investigate transition metals (e.g., Mn, Co, Fe, etc.) and their metal alloys for hydrogen splitting, because of their higher H_2_ storage capacities and low cost [5,6,7,8]. However, one of the major drawbacks of metal-based alloys is the ease of an oxide layer formation on the surfaces of these catalysts, which hinders the adsorption of the hydrogen molecule [9]. 

In catalysis, single atom catalysis (SAC) has appeared as a novel way to obtain the utmost utilization efficiency and remarkable catalytic activity. SAC is an economically viable and emerging strategy towards the maximization of catalytic efficiency, and to lower the noble metal cost [19,20,21]. Several synthetic protocols have been reported to design such SACs, such as atomic deposition layer [22], wet chemistry [23], and soft landing [24]. Various noble metal SACs such as palladium [25], platinum [26], and rhodium [27], etc., have been broadly researched to catalyze the HDR (hydrogen dissociation reaction). However, the main obstacles with such SACs are a high operating cost and temperature; therefore, these are not feasible economically [28]. Therefore, the replacement of noble metals is necessarily required with low-cost materials for large-scale and commercial applications. In this regard, transition metals (TM) such as Fe, Ni, Co, etc., have gained attention due to their high relative abundance and low price [29,30]. The performance of SACs is now taking hold; but their molecular- and electronic-level realizations are still limited. Therefore, the electronic catalytic investigation of such single atom-supported electrocatalysts is highly capitative [31].

Although significant research has been performed on abundantly present transition metals, these catalysts suffer from a high dissociation barrier and low stability issues [32]. The stability of SACs is mainly based on the support material (adsorbent). Carbon-based materials such as graphene [33,34], metal organic frameworks [35], graphitic carbon nitride [36,37], and other nanostructured surfaces have gained interest as adsorbent or support materials for SACs due to a large surface area, a promising hydrogen storage ability, and a high thermal stability [38,39,40]. Recently, the hydrogen dissociation reaction has been studied on a less expensive and abundant single transition metal atom (Fe, Co, and Ni)-doped C_2_N surface via density functional theory [41]. However, scientists are still looking for more rational and highly efficient catalytic adsorbent materials with higher efficiencies. 

Zigzag molecular nanobelts have recently captured the interest of scientists because of their appealing aesthetic structures, intriguing chemical reactivities, and tantalizing features [42]. The cylindrical, well-defined cavities of variable sizes, and the promising electronic characteristics declare zigzag molecular belts as being unique and eminent support materials, and macrocyclic hosts (supramolecular chemistry) [43]. Additionally, the hydrocarbon skeletons of these molecular belts can be considered as the smallest chunk of the zigzag single-walled CNT [44]. These molecular belts can also be viewed as potential templates to support the growth of uniform and structurally well-defined CNTs, which find their practical applications in nanoscience and advanced nanotechnology [43]. The revival interest in hydrocarbon molecular belts has been witnessed in recent years [45]. These zigzag low-coordinated molecular nanobelts have been successfully synthesized and reported in the literature to exist independently. The successful synthesis reported by Cheung et al. involves a repetitive Diels–Alder reaction, followed by the reductive aromatization of O_2_-bridged moieties [46]. Similarly, the facile reduction of ketone with *n*-butyllithium (nucleophilic addition) and NaBH_4_ produced tertiary and secondary alcohol-containing nanobelts, respectively. The selective oxidation of biscarbonyl-bearing octahydrobelt[8]arene with (PhSeO)_2_O and *m*-CPBA furnished the corresponding 1,4-quinone and lactone-embedded molecular nanobelts [47]. The molecular belt[n]arenes bring the realization of a high intrinsic conductivity, abundant catalytic active sites, a large surface area, and strong molecular adsorption. In recent times, density functional theory and experimental predictions have unveiled that catalysts with multiple active sites, including nitrogen atom or/and transition metal (TM) doping (TM = Co, Cr, Fe, Cu, Ni, or Zn) on a heterostructural electrocatalyst are more promising candidates to promote the hydrogen adsorption and hydrogen evolution process [48,49,50,51]. To the best of our knowledge, zigzag molecular nanobelts have not yet been explored for their catalytic and adsorbent capabilities. In the current study, molecular belts are utilized as adsorbents for transition metal atoms to design highly efficient electrocatalysts for the hydrogen dissociation reaction, which could provide stability, higher selectivity, and economical feasibility. 

Herein, we have chosen the hydrogen dissociation reaction (HDR) as a probe reaction to explore the catalytic performances of ten transition metals, doped H_6_-N_3_-belt[6]arene nanobelt (NB) as SACs. Currently, density functional theory (DFT)-based quantum chemical simulations are scrutinized as a highly fruitful approach towards investigating the nature and efficiency of a catalyst [52,53,54,55]. Therefore, the density functional quantum chemical method is accessed to comprehensively probe the splitting of the hydrogen molecule on the transition metal-doped H_6_-N_3_-belt[6]arene nanobelt (TM@NB) as SACs to evaluate the catalytic activities of these complexes. Moreover, the considered TM@NB complexes are also explored for thermodynamic stability, electronic features, hydrogen adsorption capacity, and the catalytic performance for HDR. 

## 2. Methodology

In the current work, density functional theory (DFT) simulations are carried out to investigate the hydrogen dissociation reaction (HDR) over various transition metal-doped nanobelts (TM@nanobelt). All the DFT calculations are simulated using the Gaussian 09 package [56], whereas the obtained results are analyzed via Gaussview 5.0 and Chemcraft [57,58,59,60]. ωB97XD, a DFT functional, is used with appropriate split valence basis sets 6-311G (d,p) to simulate the geometric and thermodynamic parameters. The wB97XD functional employees the D2 dispersion Grimme’s model, which is considered as the latest version, which includes empirical dispersion by Head–Gordon et al. [61]. The ωB97XD method is a well reported hybrid functional that mainly accounts for the accurate investigation of dispersion forces and long-range corrections [62,63,64]. wB97XD has been considered as an accurate functional in predicting molecular geometries. Moreover, the ωB97XD functional is also considered as the most appropriate functional for the geometric optimization of organic molecules [65,66,67]. In contrast, the 6-311G(d,p) basis set is chosen, which is a Pople-type basis set that contains the polarization function. The level of theory (ωB97XD/6-311G(d,p)) chosen here is well reported in literature, where it is used to investigate the potential of single-atom catalysis [68]. The frequency analysis is also performed to confirm the nature of the stationary points, such that the absence of the negative frequency validates the true minima nature of the reactants and products (stationary points) of all the studied TM@nanobelt analogues. Additionally, the presence of single imaginary frequency corroborates the presence of the transition state. Moreover, transition states are also confirmed through the motion of the reaction coordinates via the associated eigenvectors [69]. The whole first-row TM from Sc to Zn are doped over nanobelt, and their catalytic performance is investigated for the hydrogen dissociation reaction. The interaction energy (*E*_int_) analysis is performed to evaluate the mode of interaction of all the designed TM@nanobelt complexes by using Equation (1):

Δ𝐸_int_ = 𝐸_TM@NB_ − (𝐸_NB_+𝐸_TM_) (1)

Here, *E*_TM@NB_ refers to the energy of the metal-doped complex, whereas *E*_NB_ and *E*_TM_ represent the energies of individual nanobelt and transition metal (with the most stable spin state), respectively. Moreover, frontier molecular orbitals (FMOs) analysis is performed on the designed TM-doped nanobelt catalysts to see the electronic level perturbations. 

Similarly, adsorption energies (∆*E*_ads_) for the adsorption of the hydrogen molecule are also computed by using Equation (2) for all the doped nanobelts. In Equation (2), *E*_H2TM@NB_ presents the total energy of the hydrogen molecule-adsorbed TM@NB complex. *E*_TM@NB_ refers to the energy of metal-doped nanobelts, and *E*_H2_ corresponds to the energy of the isolated H_2_ molecule.

Δ𝐸_ads_ = 𝐸_H2TM@NB_ − (𝐸_TM@NB_+𝐸_H2_) (2)

The energy barriers (*E*_a_) and reaction energies (∆*E*) are estimated according to Equations (3) and (4), respectively. In Equations (3) and (4), the *E*_TS_, *E*_R_, and *E*_P_ correspond to the energies of the transition state (TS), reactants (R), and the final state or product (P), respectively. The hydrogen dissociation reaction mechanism and its pathways are evaluated by comparing the energy barriers (*E*_a_) [70].

𝐸_a_ = 𝐸_TS_ − 𝐸_R_ (3)

∆𝐸 = 𝐸_P_ − 𝐸_R_ (4)

To obtain further insight into the donor–acceptor interactions during the dissociation of the hydrogen molecule over the doped complexes, NBO and EDD analyses are also computed. For the EDD analysis, Multiwfn software is employed [71]. 

## 3. Results and Discussion

### 3.1. Geometry Optimization and Adsorption Energy

**Figure 1:**
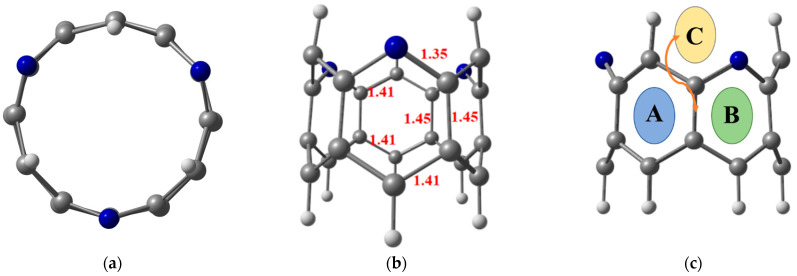
Top view (**a**) and side view (**b**) of the optimized structure of nanobelt at ωB97XD/6-311G(d,p) level of theory, where (**c**) presents the possible sites for doping transition metal atom; benzene ring (A), pyridine ring (B) and C—C bridge (C). Grey, whitish, and blue balls represent carbon, hydrogen, and nitrogen atoms, respectively.

Prior to hydrogen molecule adsorption, the designed TM@NB complexes are fully relaxed to their preferable stable geometries at the ωB97XD/6-311G(d,p) level of theory. The titled zigzag molecular nanobelt of interest, H_6_-N_3_-belt[6]arene, consists of six six-membered fused alternative benzene and pyridine rings [42]. The top and side view of the optimized structure of H_6_-N_3_-belt[6]arene with the important bond distances mentioned are given in Figure 1. The average bond distance of the edge C—C bond length is 1.41 Å, whereas the C—C bond length of the C—C bonds present at the center of fused rings is extended slightly to 1.45 Å, which is consistent with the reported bond distances [72]. Moreover, 1.35 Å of bond length is observed for all C—N bonds (see Figure 1). In this study, the first-row transition metal (TM) atoms (Sc to Zn) are adsorbed over the H_6_-N_3_-belt[6]arene nanobelt (NB) at various sites, i.e., on the top of a benzene (A) or pyridine ring (B), and over the bridge head (C). In all the studied TM@NB complexes, the bridge head site is not stable. The early transition metal complexes reveal pyridine’s top position as the most stable site for doping, whereas the late transition metal complexes show comparable stability for both the benzene and pyridine top positions. Therefore, transition metal doped over the pyridine top configuration is selected for discussion afterwards. DFT spin-polarized simulations are performed for the first four spin states of the considered TM@NB complexes, to obtain the most stable spin state (the lowest possible geometry). Among the studied spin states, a doublet is observed as the stable spin state for Sc@NB, Co@NB, and Cu@NB, whereas it is a triplet for Ti@NB and Cr@NB. Similarly, a quartet is calculated as the most stable spin states for V@NB and Mn@NB, respectively. Moreover, a quintet is the most stable spin state for Fe@NB, whereas it is a singlet for Ni@NB and Zn@NB. The TM@NB complexes with the most stable spin states are employed in this study for discussion hereafter.

**Figure 2:**
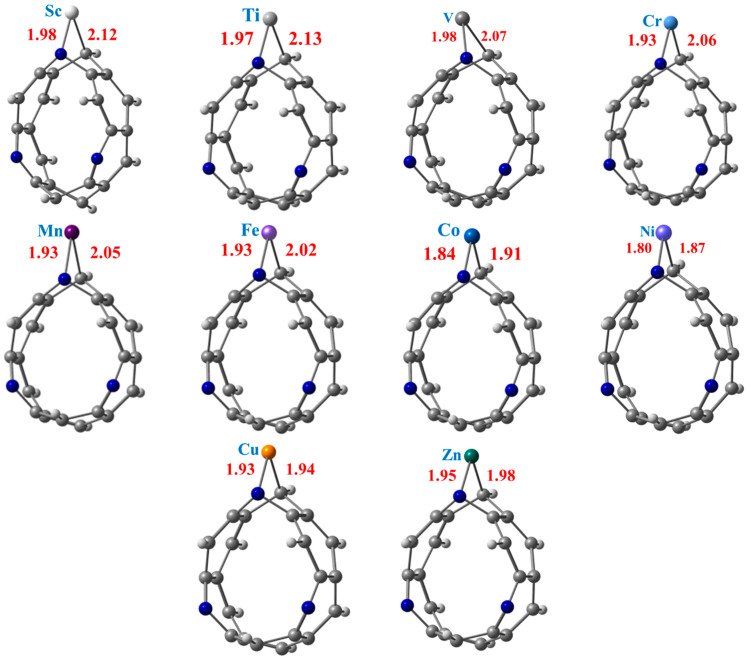
Optimized structures of stable TM@NB complexes (TM = Sc-Zn) at ωB97XD/6-311G(d,p) level of theory. Where grey, whitish, and blue balls represent carbon, hydrogen, and nitrogen atoms, respectively.

The ground state fully relaxed geometries of the TM@NB complexes with the C—TM and N—TM bond lengths mentioned, are presented in Figure 2, while their corresponding computed interaction energy (Δ*E*_int_) values are summarized in Table 1. Bond interaction distances and interaction energies are two crucial parameters to estimate the stability of a system. Therefore, it can be seen from Figure 2 that the N—TM interaction distances between the N atom pyridine ring and the doped transition metal are calculated in the range of 1.80 Å to 1.98 Å. Similarly, the C—TM bond distances between the C atom of the nanobelt and the transition metal are computed in the range of 1.87 Å to 2.13 Å in the studied complexes. The lowest C—TM and N—TM interaction distances are calculated in the case of the Ni@NB complex (see Figure 2), followed by the Co@NB and Cu@NB complexes. The highest N—TM and C—TM bond distances are observed for Sc@NB (1.98 Å) and Ti@NB (2.13 Å) among all the considered complexes, respectively. A monotonic decrease in interaction distances is seen from the Sc- to Ni-doped complexes with an increase in atomic number. However, a slight increase in the N—TM and C—TM bond distances are calculated for the Cu@NB and Zn@NB complexes. The calculation of C—TM interaction distances between the carbon atom of the nanobelt and the doped metal atom are nearly comparable with previously reported bond lengths [73]. 

Additionally, the interaction energies of the TM@NB complexes are also calculated by using Equation (1), and the values are presented in Table 1. The interaction energies with a negative sign reveal that the doping of the transition metal over the H_6_-N_3_-belt[6]arene nanobelt is a feasible process for all the considered metal atoms, i.e., Sc to Zn. Among the studied TM@NB complexes, the highest thermodynamic stability is displayed by the Ni@NB complex with the highest value of interaction energy (−4.97 eV), followed by Co@NB (−4.59 eV) and Mn@NB (−4.38 eV). Overall, the *E*_int_ values are calculated in the range of −0.18 eV to −4.97 eV for all of the considered TM@NB complexes. The lowest value of interaction energy (−0.18 eV) is observed for the Zn@NB complex, which shows that Zn atom doping over the H_6_-N_3_-belt[6]arene nanobelt is not much of a facile process. The lower value of the interaction energy for Zn atom doping over a graphene surface is also reported, which declares that Zn atom doping is not a feasible process [34]. The interaction energy results further reveal that all the doped systems are showing chemisorption, consistent with the interaction distances except for Zn (physisorption). The highest interaction energy in the case of the Ni@NB complex is also consistent with the shortest interaction distances. Overall, upon optimization, no substantial structural deformation is noticed; however, the H_6_-N_3_-belt[6]arene nanobelt structure slightly changes its shape from circular to oval upon doping (see Figure 1 and Figure 2). 

### 3.2. Electronic Properties of TM@NB Complexes

To further explore the electronic characteristics and the interactions of transition metals with the H_6_-N_3_-belt[6]arene nanobelt, natural bond orbital analysis is carried out with stable spin states. It is obvious from the electropositive nature of metal atoms, that the transition metal should transfer its charge to the H_6_-N_3_-belt[6]arene nanobelt in the designed complexes. The calculated values of the NBO charge are reported in Table 1, and the values reveal a positive charge on the metal atom, which confirms the transference of charge from metal atom to nanobelt in all of the studied TM@NB complexes. The highest NBO charge transfer is calculated in the case of the Zn@NB complex (1.241 |e|), followed by the Sc@NB (1.211 |e|), Ti@NB (1.131 |e|), and V@NB (1.124 |e|) complexes. The least NBO charge transfer is calculated in the case of Ni@NB (0.779 |e|). The maximum NBO charge transfer for the Zn@NB complex may be due to a stable d_10_ configuration after losing one electron. Overall, the amount of charge transfer for the transition metal atom in the studied TM@NB complexes is calculated in the range of 0.779 |e| to 1.241 |e|. There is a gradual decrease in the amount of NBO charge transfer with the increase in atomic number from Sc to Ni. NBO analysis strongly correlated with the interaction distances and confirms the electropositive nature of the transition metal atoms. 

**Figure 3:**
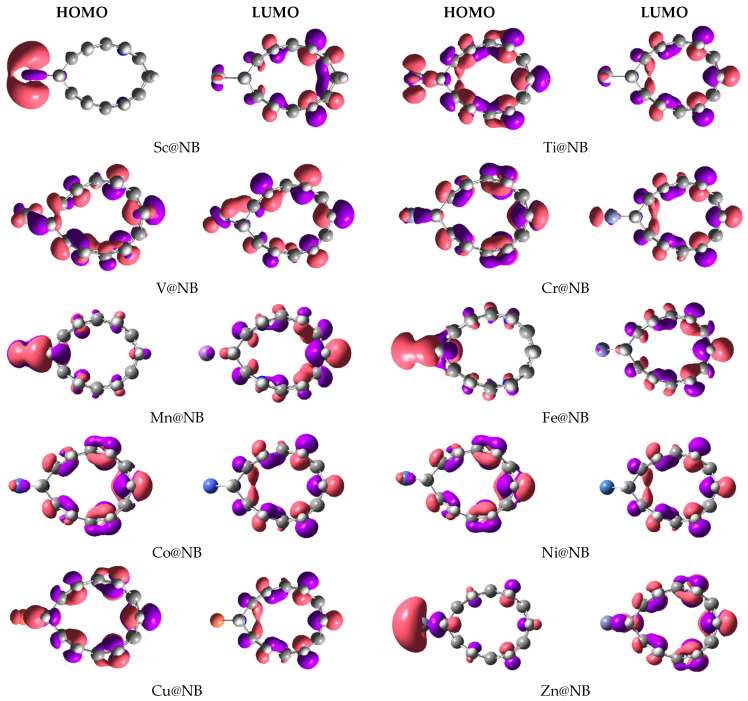
HOMO-LUMO orbital density distribution of studied TM@NB complexes (TM = Sc-Zn).

Frontier molecular orbitals analysis is also computed to visualize the electronic contributions (HOMO–LUMO isodensities), and to compute the corresponding energies. FMO analysis helps to comprehend the perturbations in the electronic properties of the doped transition metal on the H_6_-N_3_-belt[6]arene nanobelt. The HOMO–LUMO isosurfaces generated via Gaussview are given in Figure 3, and their corresponding HOMO, LUMO energies, and H-L energy gaps are summarized in Table 1. The H-L energy gap (E_g_) of the pure H_6_-N_3_-belt[6]arene nanobelt is 5.66 eV, where the values of the HOMO and LUMO energy levels are −6.94 eV and −1.28 eV, respectively. The results reported in Table 1 for FMO analysis show a reduction in the H-L energy gap in all studied TM@NB complexes, except the V@NB complex, where the energy gap remains the same (5.66 eV). The highest reduction in the energy gap compared to the bare nanobelt is seen in the case of the Zn@NB catalyst (4.76 eV), followed by Cr@NB (5.11 eV), whereas the lowest reduction in energy gap is observed for V@NB (5.66 eV) and Co@NB (5.62 eV). The FMO analysis reveals the changes in electronic properties upon doping of the transition metal atom. 

Moreover, the orbital distributions in Figure 3 reveal that the HOMO is densely occupied over the transition metal atom in most cases. The LUMO orbital density is mainly occupied over the H_6_-N_3_-belt[6]arene nanobelt. The LUMO isodensity is almost missing over the transition metal atom, except for the V@NB and Cr@NB complexes. The HOMO–LUMO orbital density distribution confirms the transfer of charge from the electropositive transition metal to the H_6_-N_3_-belt[6]arene nanobelt (MLCT) upon excitation from HOMO to LUMO. The depicted density distribution is more clear in the case of the Sc@NB, Mn@NB, Fe@NB, and Zn@NB complexes. 

### 3.3. Hydrogen Molecule Adsorption over TM@NB Complexes

**Figure 4:**
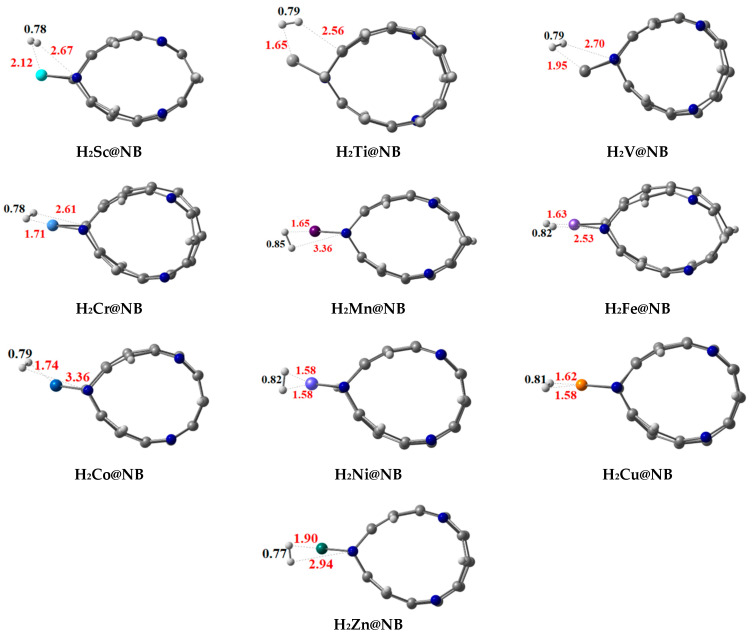
Optimized structures of stable hydrogen molecule-adsorbed TM@NB complexes (TM = Sc-Zn) at a ωB97XD/6-311G(d,p) level of theory.

The H—H bond distances (D_H-H_) for the hydrogen-adsorbed TM@NB complexes are observed in the range of 0.75 Å to 0.85 Å (labeled in black see Figure 4). Adsorption energy (*E*_ads_) is also computed by using Equation (2) for the adsorption of hydrogen molecule over the designed TM@NB complexes (see Table 2). In certain complexes, the H—H bond length of adsorbed hydrogen, the strong TM—H bond interactions, thus results in the weakening of the H—H bond. All the considered TM@NB complexes are energetically favorable for the hydrogen adsorption process, and the computed values of adsorption energy are observed in the range of −0.06 to −0.93 eV. Among all the complexes, the Co@NB complex has the highest adsorption energy value of −0.93 eV for the adsorption of the hydrogen molecule, owing to a higher thermal stability of this complex. Moreover, the Ni@NB (−0.89 eV), Cr@NB (−0.77 eV), Cu@NB (−0.72 eV), and Mn@NB (−0.54 eV) complexes have manifested higher adsorption energy values. A higher value of adsorption energy reveals a stronger binding of the hydrogen molecule over the TM@NB complexes.

Important interaction bond distances (TM—H and N—H) are also mentioned in Figure 3, which reveal that these interaction distances also vary in different doped systems. The N—H bond distances in various studied systems are observed in the range of 2.53 Å to 3.36 Å, whereas the TM—H bond distances are in the range of 1.58 Å to 2.12 Å. Overall, when a hydrogen molecule is adsorbed over the TM@NB complex, the H–H bond length is slightly increased compared to the isolated hydrogen molecule. Thus, this reveals the activation of the hydrogen molecule over the metal-doped H_6_-N_3_-belt[6]arene nanobelt. A gradual increase in H—H bond distance is observed from Sc to Mn, with an increase in atomic number upon adsorption over the TM@NB complexes, whereas the H—H bond distance decreases after Mn with an increase in atomic number, which may be due to less participation of d-orbital electrons due to the pairing up of these electrons. The lowest H—H bond distance is obtained in the case of the Zn@NB complex due to a stable d_10_ configuration.

### 3.4. Dissociation of the Hydrogen Molecule over TM@NB Complexes

**Figure 5:**
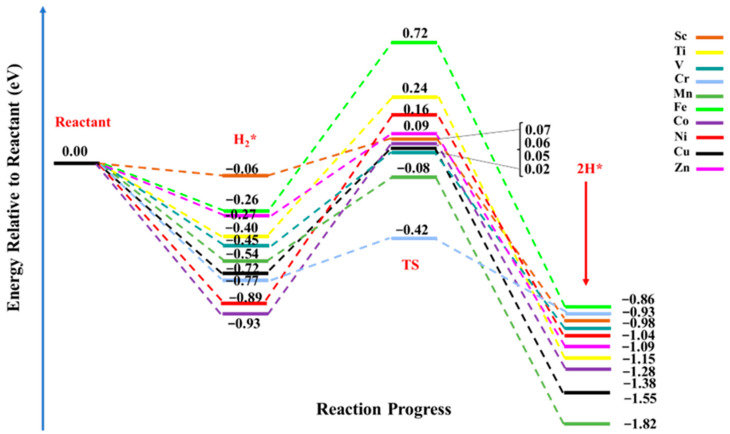
Hydrogen dissociation reaction pathway over TM@NB (TM = Sc-Zn) catalysts; all energy values are expressed in eV, and the energies of intermediate state (H_2_*), transition state (TS), and final state or products (2H*) are relative to the reactants.

The dissociative adsorption of the H_2_ molecule is considered to be one of the important reactions over catalytic surfaces. The hydrogen dissociation reaction involves the splitting of a molecular covalent bond, and at the same time, it requires the formation of new chemical bonds. Thus, an efficient catalyst is required to perform an HDR reaction. Herein, we considered TM@NB catalysts to evaluate their catalytic performances for the hydrogen dissociation reaction. In such reactions, the efficiency of the catalyst can be examined via the activation energy (*E*_a_) or the energy barrier, which is regarded as an important criterion to localize or to regulate the transfer of electrons (charged particles) [74]. Therefore, a lowering of the energy barrier facilities the transport of charges from the oxidative site (the electron donor site) to the reductive site (the electron acceptor site). The free energy diagram of the HDR pathway on the designed TM@NB catalysts is demonstrated in Figure 5. The negative values of energies declares that both the intermediate (H_2_*) and product (2H*) states are thermodynamically favorable in all TM@NB catalysts, showing the stability of these complexes. The electrocatalytic dissociation reaction of the hydrogen molecule started with the adsorption of the hydrogen molecule, followed by the heterolytic cleavage of the H—H bond, and finally, the diffusion of dissociated hydrogen atoms to their corresponding binding sites. Among the studied TM@NB catalysts, the smallest activation barrier for HDR is observed in the case of the Sc@NB catalyst (0.13 eV), while the highest activation barrier is seen in the case of the Ni@NB catalyst (1.05 eV). Moreover, the lowest dissociation barrier of the Sc@NB complex is followed by the Cr@NB, Zn@NB, Mn@NB, and V@NB catalysts, with energy barriers of 0.35 eV, 0.36 eV, 0.46 eV, and 0.47 eV, respectively. The dissociation barrier for the Ti@NB catalyst is quite close to the Cr@NB catalyst, with a value of 0.65 eV. While for rest of the studied metal-doped H_6_-N_3_-belt[6]arene nanobelt catalysts, the hydrogen dissociation barriers are 0.77 eV, 0.98 eV, 0.99 eV, and 1.33 eV for Cu@NB, Fe@NB, Co@NB, and Ni@NB, respectively. At a transition state, the H—H bond lengths vary between 0.89 Å and 1.07 Å, which shows that the H—H bond length increases in all of the studied complexes, compared to their corresponding intermediate counterparts. Similarly, the N—H and TM—H bond distances are decreased at the transition state (TS), as compared to the intermediate (H_2_*) state.

**Figure 6:**
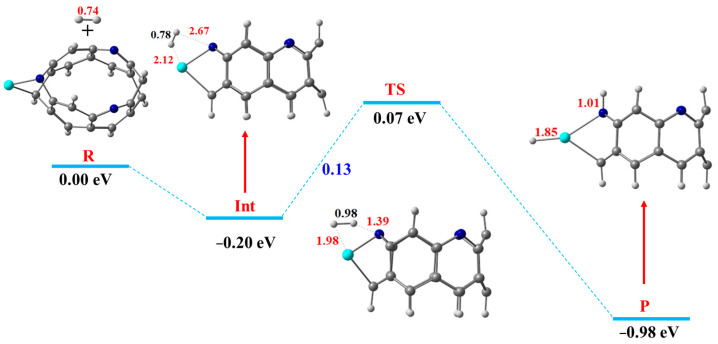
The free energy diagram of the HDR mechanism over the Sc@NB catalyst, where the reactant, intermediate, transition state, and product are demonstrated as R, Int, TS, and P (H, light grey; C, grey; Sc, cyan blue; N, blue).

Herein, the best catalyst efficiency for the hydrogen dissociation reaction is obtained for Sc@NB catalyst; therefore, the HDR over the Sc@NB catalyst is taken as a representative model to discuss the reaction mechanism in detail. The detailed free energy diagram of the hydrogen dissociation reaction mechanism over the Sc@NB complex, along with the important bond lengths mentioned, is presented in Figure 6. Initially, the hydrogen molecule (reactant) becomes adsorbed over the Sc@NB catalyst from the side edge position, directly approaching towards the transition metal atom (Sc) and the nitrogen atom of the nanobelt, as shown in Figure 6. The hydrogen dissociation reaction (HDR) H_2_* → 2H* resulted in the dissociation of molecular hydrogen into two atomic hydrogens, which are diffused to the nitrogen site and the metal active site on the product side. This molecular hydrogen dissociation process requires overcoming the dissociation energy barrier of 0.13 eV (3.00 kcal/mol). In dissociation reactions, the H−H bond length is considered as a crucial structural parameter, which predicts the stabilities of intermediate and transition states. At the transition state, the H—H bond length extends to 0.98 Å, which was primarily at 0.78 Å in an intermediate state (the hydrogen adsorbed Sc@NB complex). This substantial elongation in H—H bond distance at the transition state compared to the intermediate state declares the facilitated activation of the H_2_ molecule over the Sc@NB catalyst. Figure 6 shows that the splitting of the hydrogen molecule is proceeded through heterolytic cleavage, where the hydrogen is dissociated into the hydride ion and a proton. The N—H and TM—H interaction bond distances at the transition state are decreased to 1.39 Å and 1.98 Å from 2.67 Å and 2.12 Å (intermediate state), respectively. After dissociation, the hydride ion diffuses towards a metal atom (Sc), while the proton diffuses towards a nitrogen atom of the H_6_-N_3_-belt[6]arene nanobelt. The molecule hydrogen dissociation is accompanied by the evolution of −1.05 eV of heat. Moreover, the Sc—H and N—H bond distances upon the binding of hydrogen atoms are 1.85 Å and 1.01 Å, respectively. Furthermore, the enthalpy of the H_2_Sc@NB complex (product side 2H*) is −0.98 eV, thus showing that the dissociated hydrogen (product) is more stabilized compared to molecular hydrogen (reactant).

Table 2 reveals that the dissociation barriers over various designed TM@NB catalysts for the hydrogen dissociation reaction are observed in the broad range of 0.13 eV to 1.05 eV. Figure 5 further shows that the products are thermodynamically more stabilized than their corresponding reactants. Among all the considered catalysts, the smallest hydrogen dissociation barrier is seen for the Sc@NB complex (0.13 eV), which can be readily attained under ambient conditions (a prerequisite for the hydrogenation reaction). This declares that the Sc@NB complex has the most efficient catalytic activity for HDR in comparison to the rest of the studied TM@NB complexes. Similarly, the second-best catalytic activity for the hydrogen dissociation reaction is observed for the Zn@NB catalyst (0.36 eV). The observed catalytic efficiency for the Sc@NB (0.13) catalyst is remarkably better than the already reported metal-based surfaces (Mg_15_Ni_2_Al_12_) for the hydrogen dissociation reaction, where the dissociation barrier was 0.82 eV [75]. Similarly, a dissociation barrier over designed catalysts is even better then what has been reported for the noble metal atom-based single atom catalyst (CeO_2_ surface), where the barrier is in the range of 0.30 eV to 0.99 eV [24]. The Sc@NB catalyst delivers outstanding catalytic activity for HDR; therefore, the Sc@NB catalyst can be utilized as a promising and cost-effective single atom-based catalyst for hydrogenation reactions.

### 3.5. NBO and EDD Analyses 

The mechanism of hydrogen molecule activation and splitting over the designed complexes is further elaborated through charge transfer via natural bond orbital and electron density difference analyses. Natural bond orbital (NBO) analysis helps to estimate the amount of NBO charge transfer that occurs from transition metals (d-orbitals) upon the adsorption of the hydrogen molecule. The computed amount of NBO charges on hydrogen atoms, nitrogen atoms, and transition metal atoms of H_2_-adsorbed TM@NB complexes are summarized in Table 3. In all of the H_2_TM@NB complexes, the hydrogen molecule dissociated into hydride ion and a proton, which after dissociation, is stabilized over the transition metal atom and the nitrogen atom of the nanobelt, respectively. Hence, NBO analysis reveals that TM atoms exhibit a positive NBO charge, while the hydrogen atom (H1) interacting with TM has a negative NBO charge, which confirms the transfer of charge from the metal atom to the hydrogen atom. Similarly, the NBO charge on the nitrogen atom is negative, whereas on the corresponding hydrogen atom (H2), a positive NBO charge is seen. NBO analysis displays the electropositive nature of transition metals, therefore transferring the NBO charge to the H atom. The maximum NBO charge transfer is obtained for the Sc metal atom (+1.444 |e|), whereas the minimum charge transfer is seen for the Ni metal (+0.916 |e|); the trend is consistent with the previously discussed trend in Section 3.1 and Section 3.2.

**Figure 7:**
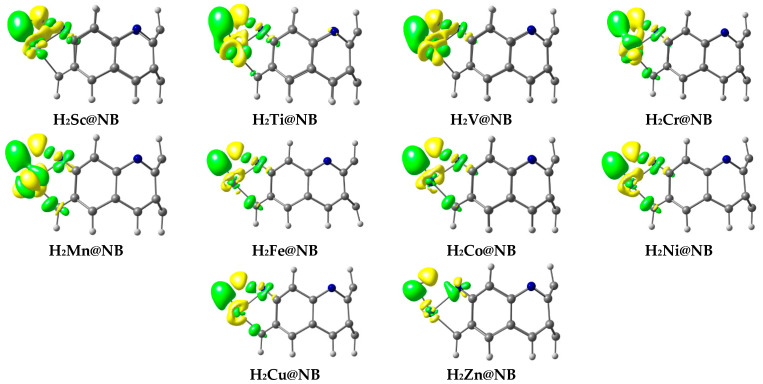
EDD analysis of hydrogen-adsorbed TM@NB complexes; green color is for electron density accumulation, whereas yellow color is for depletion of electronic density.

EDD is performed to validate the NBO results, and the plotted EDD isosurfaces for the designed hydrogen-adsorbed TM@NB complexes are given in Figure 7. The green and yellow isosurfaces are seen in the EDD plots; this therefore corroborates the charge transfer upon hydrogen dissociation. Green colored isosurfaces present the accumulation of charge density, whereas the yellow colored isosurfaces are showing the depletion of charge density. From the EDD plots, it is obvious that a transfer of charge occurs from the transition metal (TM) to the hydrogen atom (H1), and from the hydrogen atom (H2) to nitrogen (N) upon hydrogen dissociation in all of the considered TM@NB complexes (for details, check Figure 7). In all TM@NB complexes, the yellow colored isosurfaces primarily appear over the TM atoms and hydrogen atoms interacting with the nitrogen atom of the molecular nanobelt presenting the depletion of charge density. Similarly, green colored patches appear over the hydrogen atom (H1) and the nitrogen atom of the nanobelt, displaying the accumulation of electronic charge density. Overall, the EDD results show a strong correlation with the results of the NBO charge transfer analysis. Additionally, the process of charge transfer facilitates the filling of electrons in the σ* (antibonding orbital) of the H_2_ molecule, hence making the dissociation process feasible over TM@NB catalysts. 

For comparison, the activation barrier of hydrogen dissociation in our work (Sc@NB catalyst) and some other reported surfaces are summarized in Table 4. Our computed value of the hydrogen dissociation barrier for the Sc@NB catalyst (0.13 eV) declares a highly efficient performance of our designed catalysts with respect to the reported ones. In our case, the least activation barrier is calculated for the Sc@NB catalyst, and the catalytic performance is much better, even than the reported Au/TiO2 catalyst (0.54 eV).

The computational simulations are not always reflecting the real-world conditions, and the results obtained may theoretically have differences from those of the experimental results. These differences arise due to the limitations of the computational methods, but it is worth mentioning that the computational results provide very useful guidelines for experimentalists, and the trends obtained theoretically match very much with experimental values (in most cases), although the absolute values may have differences. Several studies have been published where the results obtained theoretically for mechanistic studies corroborate nicely with the experimental results [78,79,80,81,82]. 

The most crucial factor in a computational study is the choice of the level of theory because an accurate level of theory can lead to reliable results. There are several theoretical methods that are available in the literature, and many times, it becomes quite difficult to choose a functional for theoretical study. Therefore, calibration is needed before a functional (or method) can be chosen. Many such benchmark studies are available in the literature, where functionals from a variety of classes are evaluated against the experimental data or against data from a higher level of theory. We have taken assistance from the literature benchmark studies for selecting the chosen functional. This is since weak intermolecular forces are believed to be involved in this system when hydrogen is dissociating on the metal-doped nanobelts, and the best functional for describing these weak non-bonding interactions is wB97XD [83]. With a properly chosen functional, the errors in the activation barriers are roughly ±1.5 kcal/mol. Since a calibrated method is chosen, we are therefore very confident that the results obtained are within the ±1.5 kcal/mol error limit [84]. However, despite these errors, the trends obtained theoretically are generally consistent with the trends obtained experimentally.

## 4. Conclusions

In conclusion, we performed a systematic DFT investigation on the adsorption and dissociation process of the hydrogen molecule over TM-doped H_6_-N_3_-belt[6]arene nanobelt-based single-atom catalysts. The doping of transition metal (TM) over nanobelt is an exothermic process for all of the studied metals, and a maximum interaction energy (*E*_int_) is obtained for the Ni@NB complex (−4.97 eV). NBO analysis reveals the electropositive nature of the metal atoms, and the results are consistent with FMO outcomes. Additionally, the adsorption energy (*E*_ads_) for the adsorption of the hydrogen molecule over the designed TM@NB complexes, and their corresponding dissociation barriers are also computed. The reaction mechanism pathway reveals that the 2H* state (atomic hydrogen) is thermodynamically more favorable due to a negative energy level (exothermic process) in all of the studied complexes. The minimum dissociation barrier is seen for the Sc@NB complex (0.13 eV), followed by the Zn@NB complex (0.36 eV) among all of the studied TM@NB catalysts. The NBO charge transfer analysis shows that charge is being transferred from the transition metal to the H_2_ molecule, thus facilitating the process of hydrogen dissociation. Moreover, for a visual depiction of charge transfer, EDD analysis is also performed, which shows an agreement with NBO analysis. In summary, this study reveals the Sc@NB catalyst as the most effective catalyst for the hydrogen dissociation reaction, and hence it paves a way for experimentalists to engineer efficient and less expensive electrocatalysts for the hydrogen dissociation process. Moreover, the current study also provides a new avenue for material scientists to design SACs based on other nanobelts with second- and third-row transition metals for the facile hydrogen dissociation process. Furthermore, a transition metal-doped H_6_-N_3_-belt[6]arene nanobelt-based catalyst might be applicable to catalyze different chemical transformations.

## Figures and Tables

**Table 1:** Summary of energies for the interaction of TM atom over nanobelt (Δ*E*_int_), HOMO (eV), LUMO (eV), energy gap (eV), and NBO charges on TM atom of all considered TM@NB complexes.

Complexes	Δ*E*_int_ (eV)	HOMO (eV)	LUMO (eV)	E_g_ (eV)	Q_TM_ |e|
Sc@NB	−3.54	−6.06	−0.52	5.53	1.211
Ti@NB	−3.57	−6.28	−0.86	5.42	1.131
V@NB	−3.31	−6.47	−0.81	5.66	1.124
Cr@NB	−3.68	−6.42	−1.31	5.11	1.097
Mn@NB	−4.38	−5.33	−2.10	3.23	0.991
Fe@NB	−2.10	−6.20	−1.07	5.13	0.971
Co@NB	−4.59	−6.41	−0.79	5.62	0.939
Ni@NB	−4.97	−6.34	−0.89	5.45	0.779
Cu@NB	−3.01	−6.27	−0.95	5.32	0.939
Zn@NB	−0.18	−6.20	−1.44	4.76	1.241
Nanobelt (NB)	--	−6.94	−1.28	5.66	--

**Table 2:** Summary of hydrogen molecule adsorption energies (Δ*E*_ads_), energy of reaction (ΔE), and hydrogen dissociation energy barriers (*E*_a_) for all studied TM@NB complexes.

Complexes	Δ*E*_ads_ (eV)	ΔE	*E*_a_
H_2_Sc@NB	−0.06	−0.98	0.13
H_2_Ti@NB	−0.40	−1.28	0.65
H_2_V@NB	−0.45	−1.04	0.47
H_2_Cr@NB	−0.77	−0.93	0.35
H_2_Mn@NB	−0.54	−1.82	0.46
H_2_Fe@NB	−0.26	−0.86	0.98
H_2_Co@NB	−0.93	−1.38	0.99
H_2_Ni@NB	−0.89	−1.09	1.05
H_2_Cu@NB	−0.72	−1.55	0.77
H_2_Zn@NB	−0.27	−1.15	0.36

**Table 3:** NBO charge transfer analysis of stable H_2_TM@NB complexes.

Complexes	H1 (TM Side) |e|	TM (|e|)	H2 (N Side) |e|	N (|e|)
H_2_Sc@NB	−0.257	1.444	0.244	−0.764
H_2_Ti@NB	−0.315	1.254	0.252	−0.809
H_2_V@NB	−0.284	1.106	0.252	−0.774
H_2_Cr@NB	−0.315	1.235	0.245	−0.789
H_2_Mn@NB	−0.317	1.049	0.273	−0.800
H_2_Fe@NB	−0.323	1.111	0.271	−0.755
H_2_Co@NB	−0.327	1.050	0.281	−0.743
H_2_Ni@NB	−0.309	0.916	0.295	−0.711
H_2_Cu@NB	−0.295	0.975	0.238	−0.705
H_2_Zn@NB	−0.218	1.351	0.176	−0.982

**Table 4:** Comparison of hydrogen dissociation barrier over Sc@NB catalyst with already reported barriers over various surfaces.

Catalysts for HDR	Energy Barrier (eV)	Reference
Sc@NB	0.13 eV	Our work
Fe@C_2_N	0.36 eV	Shah et al. [41]
Mg_15_Ni_2_Al_12_	0.53 eV	Zhang et al. [75]
Au/TiO_2_ system	0.54 eV	Sun et al. [76]
Ti-doped Mg Surface	0.35 eV	Du et al. [77]

## Data Availability

All data are provided in the manuscript.

## References

[1] Arsad A.Z., Hannan M., Al-Shetwi A.Q., Mansur M., Muttaqi K., Dong Z., Blaabjerg F. (2022). Hydrogen energy storage integrated hybrid renewable energy systems: A review analysis for future research directions. Int. J. Hydrogen Energy.

[2] Rosen M.A., Koohi-Fayegh S. (2016). The prospects for hydrogen as an energy carrier: An overview of hydrogen energy and hydrogen energy systems. Energy Ecol. Environ..

[3] Zhang F., Zhao P., Niu M., Maddy J. (2016). The survey of key technologies in hydrogen energy storage. Int. J. Hydrogen Energy.

[4] Yanxing Z., Maoqiong G., Yuan Z., Xueqiang D., Jun S. (2019). Thermodynamics analysis of hydrogen storage based on compressed gaseous hydrogen, liquid hydrogen and cryo-compressed hydrogen. Int. J. Hydrogen Energy.

[5] Niaz S., Manzoor T., Pandith A.H. (2015). Hydrogen storage: Materials, methods and perspectives. Renew. Sustain. Energy Rev..

[6] Rusman N.A.A., Dahari M. (2016). A review on the current progress of metal hydrides material for solid-state hydrogen storage applications. Int. J. Hydrogen Energy.

[7] Zhou L., Zhang C., McClain M.J., Manjavacas A., Krauter C.M., Tian S., Berg F., Everitt H.O., Carter E.A., Nordlander P. (2016). Aluminum nanocrystals as a plasmonic photocatalyst for hydrogen dissociation. Nano Lett..

[8] Sun M., Nelson A.E., Adjaye J. (2005). Ab initio DFT study of hydrogen dissociation on MoS2, NiMoS, and CoMoS: Mechanism, kinetics, and vibrational frequencies. J. Catal..

[9] Fujitani T., Nakamura I., Akita T., Okumura M., Haruta M. (2009). Hydrogen dissociation by gold clusters. Angew. Chem. Int. Ed..

[10] Allangawi A., Gilani M.A., Ayub K., Mahmood T. (2023). First row transition metal doped B12P12 and Al12P12 nanocages as excellent single atom catalysts for the hydrogen evolution reaction. Int. J. Hydrogen Energy.

[11] Mehboob M.Y., Hussain R., Younas F., Jamil S., Iqbal M.M.A., Ayub K., Sultana N., Janjua M.R.S.A. (2022). Computation assisted design and prediction of alkali-metal-centered B12N12 nanoclusters for efficient H2 adsorption: New hydrogen storage materials. J. Clust. Sci..

[12] Mehboob M.Y., Hussain F., Hussain R., Ali S., Irshad Z., Adnan M., Ayub K. (2021). Designing of Inorganic Al12N12 Nanocluster with Fe, Co, Ni, Cu and Zn Metals for Efficient Hydrogen Storage Materials. J. Comput. Biophys. Chem..

[13] Jing S., Zhang L., Luo L., Lu J., Yin S., Shen P.K., Tsiakaras P. (2018). N-doped porous molybdenum carbide nanobelts as efficient catalysts for hydrogen evolution reaction. Appl. Catal. B Environ..

[14] Chen J.L., Zhang X.G., Wu D.Y. (2022). Dissociation reactions of hydrogen molecules at active sites on gold clusters: A DFT study. J. Chin. Chem. Soc..

[15] Tierney H.L., Baber A.E., Kitchin J.R., Sykes E.C.H. (2009). Hydrogen dissociation and spillover on individual isolated palladium atoms. Phys. Rev. Lett..

[16] van Steen E., van Helden P. (2010). A DFT study of hydrogen dissociation on CO-and C-precovered Fe (100) surfaces. J. Phys. Chem. C.

[17] Zha H., Dong X., Yu Y., Zhang M. (2018). Hydrogen-assisted versus hydroxyl-assisted CO dissociation over Co-doped Cu (111): A DFT study. Surf. Sci..

[18] Weng M.H., Chen H.-T., Wang Y.-C., Ju S.-P., Chang J.-G., Lin M.-C. (2012). Kinetics and mechanisms for the adsorption, dissociation, and diffusion of hydrogen in Ni and Ni/YSZ slabs: A DFT study. Langmuir.

[19] Liu D., Barbar A., Najam T., Javed M.S., Shen J., Tsiakaras P., Cai X. (2021). Single noble metal atoms doped 2D materials for catalysis applications. Appl. Catal. B Environ..

[20] Liang S., Hao C., Shi Y. (2015). The power of single-atom catalysis. ChemCatChem.

[21] Chen F., Jiang X., Zhang L., Lang R., Qiao B. (2018). Single-atom catalysis: Bridging the homo-and heterogeneous catalysis. Chin. J. Catal..

[22] Cheng N., Sun X. (2017). Single atom catalyst by atomic layer deposition technique. Chin. J. Catal..

[23] Chen Y., Ji S., Chen C., Peng Q., Wang D., Li Y. (2018). Single-atom catalysts: Synthetic strategies and electrochemical applications. Joule.

[24] Righi G., Magri R., Selloni A. (2019). H2 Dissociation on Noble Metal Single Atom Catalysts Adsorbed on and Doped into CeO_2_ (111). J. Phys. Chem. C.

[25] Guo Y., Lang R., Qiao B. (2019). Highlights of major progress on single-atom catalysis in 2017. Catalysts.

[26] Qiao B., Wang A., Yang X., Allard L.F., Jiang Z., Cui Y., Liu J., Li J., Zhang T. (2011). Single-atom catalysis of CO oxidation using Pt 1/FeO_x_. Nat. Chem..

[27] Ghosh T.K., Nair N.N. (2013). Rh_1_/γ-Al_2_O_3_ Single-Atom Catalysis of O_2_ Activation and CO Oxidation: Mechanism, Effects of Hydration, Oxidation State, and Cluster Size. ChemCatChem.

[28] Parkinson G.S. (2019). Single-atom catalysis: How structure influences catalytic performance. Catal. Lett..

[29] Cheng N., Zhang L., Doyle-Davis K., Sun X. (2019). Single-atom catalysts: From design to application. Electrochem. Energy Rev..

[30] Liang J.-X., Yang X.-F., Wang A., Zhang T., Li J. (2016). Theoretical investigations of non-noble metal single-atom catalysis: Ni 1/FeO_x_ for CO oxidation. Catal. Sci. Technol..

[31] Thiel W. (2014). Computational catalysis—Past, present, and future. Angew. Chem. Int. Ed..

[32] Pozzo M., Alfe D. (2009). Hydrogen dissociation and diffusion on transition metal (= Ti, Zr, V, Fe, Ru, Co, Rh, Ni, Pd, Cu, Ag)-doped Mg (0001) surfaces. Int. J. Hydrogen Energy.

[33] Ma D., Li T., Wang Q., Yang G., He C., Ma B., Lu Z. (2015). Graphyne as a promising substrate for the noble-metal single-atom catalysts. Carbon.

[34] Ullah F., Ayub K., Mahmood T. (2021). High performance SACs for HER process using late first-row transition metals anchored on graphyne support: A DFT insight. Int. J. Hydrogen Energy.

[35] Sun T., Xu L., Wang D., Li Y. (2019). Metal organic frameworks derived single atom catalysts for electrocatalytic energy conversion. Nano Res..

[36] Fu J., Wang S., Wang Z., Liu K., Li H., Liu H., Hu J., Xu X., Li H., Liu M. (2020). Graphitic carbon nitride based single-atom photocatalysts. Front. Phys..

[37] Rad A.S., Ayub K. (2016). Enhancement in hydrogen molecule adsorption on B_12_N_12_ nano-cluster by decoration of nickel. Int. J. Hydrogen Energy.

[38] Papa V., Cao Y., Spannenberg A., Junge K., Beller M. (2020). Development of a practical non-noble metal catalyst for hydrogenation of N-heteroarenes. Nat. Catal..

[39] Franco F., Rettenmaier C., Jeon H.S., Cuenya B.R. (2020). Transition metal-based catalysts for the electrochemical CO_2_ reduction: From atoms and molecules to nanostructured materials. Chem. Soc. Rev..

[40] Ayub K. (2017). Transportation of hydrogen atom and molecule through X_12_Y_12_ nano-cages. Int. J. Hydrogen Energy.

[41] Shah A.B., Sarfaraz S., Yar M., Sheikh N.S., Hammud H.H., Ayub K. (2023). Remarkable Single Atom Catalyst of Transition Metal (Fe, Co & Ni) Doped on C2N Surface for Hydrogen Dissociation Reaction. Nanomaterials.

[42] Shi T.-H., Tong S., Jiao L., Wang M.-X. (2020). A Theoretical Study on the Macrocyclic Strain of Zigzag Molecular Belts. Org. Mater..

[43] Shi T.-H., Wang M.-X. (2021). Zigzag hydrocarbon belts. CCS Chem..

[44] Zhang Y.-E., Tong S., Wang M.-X. (2021). Selective Oxidation of Belt [4] arene [4] tropilidene and Its Application to Construct Hydrocarbon Belts of Truncated Cone Structure with Expand Cavity. Org. Lett..

[45] Zhang Q., Zhang Y.-E., Tong S., Wang M.-X. (2020). Hydrocarbon belts with truncated cone structures. J. Am. Chem. Soc..

[46] Cheung K.Y., Watanabe K., Segawa Y., Itami K. (2021). Synthesis of a zigzag carbon nanobelt. Nat. Chem..

[47] Zhang Y., Tong S., Wang M.X. (2020). Synthesis and structure of functionalized zigzag hydrocarbon belts. Angew. Chem..

[48] Chandrasekaran S., Li N., Zhuang Y., Sui L., Xiao Z., Fan D., Aravindan V., Bowen C., Lu H., Liu Y. (2022). Interface charge density modulation of a lamellar-like spatially separated Ni9S8 nanosheet/Nb_2_O_5_ nanobelt heterostructure catalyst coupled with nitrogen and metal (M = Co, Fe, or Cu) atoms to accelerate acidic and alkaline hydrogen evolution reactions. Chem. Eng. J..

[49] Xu H., Jia H., Fei B., Ha Y., Li H., Guo Y., Liu M., Wu R. (2020). Charge Transfer Engineering via Multiple Heteroatom Doping in Dual Carbon-Coupled Cobalt Phosphides for Highly Efficient Overall Water Splitting. Appl. Catal. B Environ..

[50] Liu Y., Jiang S., Li S., Zhou L., Li Z., Li J., Shao M. (2019). Interface engineering of (Ni, Fe)S_2_@MoS_2_ heterostructures for synergetic electrochemical water splitting. Appl. Catal. B Environ..

[51] Wang C., Li Y., Gu C., Zhang L., Wang X., Tu J. (2022). Active Co@CoO core/shell nanowire arrays as efficient electrocatalysts for hydrogen evolution reaction. Chem. Eng. J..

[52] Huang Z.-F., Song J., Du Y., Xi S., Dou S., Nsanzimana J.M.V., Wang C., Xu Z.J., Wang X. (2019). Chemical and structural origin of lattice oxygen oxidation in Co–Zn oxyhydroxide oxygen evolution electrocatalysts. Nat. Energy.

[53] Hussain S., Shahid Chatha S.A., Hussain A.I., Hussain R., Mehboob M.Y., Gulzar T., Mansha A., Shahzad N., Ayub K. (2020). Designing novel Zn-decorated inorganic B_12_P_12_ nanoclusters with promising electronic properties: A step forward toward efficient CO_2_ sensing materials. ACS Omega.

[54] Ahmed A., Ullah H., Ullah Z., Tariq M., Ayub K. (2019). External stimulus controlled recombination of hydrogen in photochromic dithienylethene frustrated lewis pairs. Int. J. Hydrogen Energy.

[55] Sajid H., Malik S., Rashid U., Mahmood T., Ayub K. (2021). Hydrogen adsorption on Ge_52_^−^, Ge_92_^−^ and Sn_92_^−^ Zintl clusters: A DFT study. Comput. Theor. Chem..

[56] Frisch M., Trucks G., Schlegel H., Scuseria G., Robb M., Cheeseman J., Scalmani G., Barone V., Mennucci B., Petersson G. (2009). Gaussian 09, Revision D. 01.

[57] Dennington R., Keith T., Millam J. (2009). GaussView, Version 5.

[58] Sarfaraz S., Yar M., Ans M., Gilani M.A., Ludwig R., Hashmi M.A., Hussain M., Muhammad S., Ayub K. (2022). Computational investigation of a covalent triazine framework (CTF-0) as an efficient electrochemical sensor. RSC Adv..

[59] Sarfaraz S., Yar M., Ayub K. (2022). Covalent triazine framework (CTF-0) surface as a smart sensing material for the detection of CWAs and industrial pollutants. Mater. Sci. Semicond. Process..

[60] Sarfaraz S., Yar M., Khan A.A., Ahmad R., Ayub K. (2022). DFT investigation of adsorption of nitro-explosives over C_2_N surface: Highly selective towards trinitro benzene. J. Mol. Liq..

[61] Chai J.-D., Head-Gordon M. (2008). Long-range corrected hybrid density functionals with damped atom–atom dispersion corrections. Phys. Chem. Chem. Phys..

[62] Jindal R., Sharma V., Shukla A. (2022). Density functional theory study of the hydrogen evolution reaction in haeckelite boron nitride quantum dots. Int. J. Hydrogen Energy.

[63] Ans M., Iqbal J., Ahmad Z., Muhammad S., Hussain R., Eliasson B., Ayub K. (2018). Designing three-dimensional (3D) non-fullerene small molecule acceptors with efficient photovoltaic parameters. ChemistrySelect.

[64] Ans M., Iqbal J., Ayub K., Ali E., Eliasson B. (2019). Spirobifluorene based small molecules as an alternative to traditional fullerene acceptors for organic solar cells. Mater. Sci. Semicond. Process..

[65] Minenkov Y., Singstad Å., Occhipinti G., Jensen V.R. (2012). The accuracy of DFT-optimized geometries of functional transition metal compounds: A validation study of catalysts for olefin metathesis and other reactions in the homogeneous phase. Dalton Trans..

[66] Zara Z., Iqbal J., Ayub K., Irfan M., Mahmood A., Khera R.A., Eliasson B. (2017). A comparative study of DFT calculated and experimental UV/Visible spectra for thirty carboline and carbazole based compounds. J. Mol. Struct..

[67] Rad A.S., Ayub K. (2017). Adsorption properties of acetylene and ethylene molecules onto pristine and nickel-decorated Al_12_N_12_ nanoclusters. Mater. Chem. Phys..

[68] Allangawi A., Mahmood T., Ayub K., Gilani M.A. (2023). Anchoring the late first row transition metals with B_12_P_12_ nanocage to act as single atom catalysts toward oxygen evolution reaction (OER). Mater. Sci. Semicond. Process..

[69] Mukhtar A., Sarfaraz S., Ayub K. (2022). Organic transformations in the confined space of porous organic cage CC2; catalysis or inhibition. RSC Adv..

[70] Wang J., Zhao B., Liu S., Zhu D., Huang F., Yang H., Guan H., Song A., Xu D., Sun L. (2022). Catalytic pyrolysis of biomass with Ni/Fe-CaO-based catalysts for hydrogen-rich gas: DFT and experimental study. Energy Convers. Manag..

[71] Lu T., Chen F. (2012). Multiwfn: A multifunctional wavefunction analyzer. J. Comput. Chem..

[72] Kosar N., Tahir H., Ayub K., Gilani M.A., Imran M., Mahmood T. (2022). Remarkable nonlinear optical response of Mn@C_20_ (M = Na & K and n = 1–6); a DFT outcome. Mater. Sci. Semicond. Process..

[73] Zhu C., Xia H. (2018). Carbolong chemistry: A story of carbon chain ligands and transition metals. Acc. Chem. Res..

[74] Lei Y., Pakhira S., Fujisawa K., Wang X., Iyiola O.O., Perea López N.s., Laura Elías A., Pulickal Rajukumar L., Zhou C., Kabius B. (2017). Low-temperature synthesis of heterostructures of transition metal dichalcogenide alloys (W_x_ Mo_1–x_ S_2_) and graphene with superior catalytic performance for hydrogen evolution. ACS Nano.

[75] Zhang Z., Zhou X., Liu C., Guo J., Ning H. (2018). Hydrogen adsorption and dissociation on nickel-adsorbed and -substituted Mg_17_Al_12_ (100) surface: A density functional theory study. Int. J. Hydrogen Energy.

[76] Sun K., Kohyama M., Tanaka S., Takeda S. (2014). A study on the mechanism for H_2_ dissociation on Au/TiO_2_ catalysts. J. Phys. Chem. C.

[77] Du A., Smith S.C., Yao X., Lu G. (2005). The role of Ti as a catalyst for the dissociation of hydrogen on a Mg (0001) surface. J. Phys. Chem. B.

[78] Noreen M., Rasool N., Gull Y., Zubair M., Mahmood T., Ayub K., Nasim F.-u.-H., Yaqoob A., Zia-Ul-Haq M., De Feo V. (2015). Synthesis, density functional theory (DFT), urease inhibition and antimicrobial activities of 5-aryl thiophenes bearing sulphonylacetamide moieties. Molecules.

[79] Salman G.A., Nisa R.U., Iaroshenko V.O., Iqbal J., Ayub K., Langer P. (2012). Pyrrole versus quinoline formation in the palladium catalyzed reaction of 2-alkynyl-3-bromothiophenes and 2-alkynyl-3-bromofurans with anilines. A combined experimental and computational study. Org. Biomol. Chem..

[80] Ahmad G., Rasool N., Ikram H.M., Gul Khan S., Mahmood T., Ayub K., Zubair M., Al-Zahrani E., Ali Rana U., Akhtar M.N. (2017). Efficient synthesis of novel pyridine-based derivatives via Suzuki cross-coupling reaction of commercially available 5-bromo-2-methylpyridin-3-amine: Quantum mechanical investigations and biological activities. Molecules.

[81] Islam M.M., Alam M.T., Ohsaka T. (2008). Electrical double-layer structure in ionic liquids: A corroboration of the theoretical model by experimental results. J. Phys. Chem. C.

[82] Hassan J., Naz S., Haider A., Raza A., Ul-Hamid A., Qumar U., Haider J., Goumri-Said S., Kanoun M.B., Ikram M. (2021). h-BN nanosheets doped with transition metals for environmental remediation; a DFT approach and molecular docking analysis. Mater. Sci. Eng. B.

[83] DiLabio G.A., Koleini M. (2014). Dispersion-correcting potentials can significantly improve the bond dissociation enthalpies and noncovalent binding energies predicted by density-functional theory. J. Chem. Phys..

[84] Feyereisen M.W., Feller D., Dixon D.A. (1996). Hydrogen Bond Energy of the Water Dimer. J. Phys. Chem..

